# *Chromobacterium haemolyticum* Pneumonia Associated with Near-Drowning and River Water, Japan

**DOI:** 10.3201/eid2609.190670

**Published:** 2020-09

**Authors:** Hajime Kanamori, Tetsuji Aoyagi, Makoto Kuroda, Tsuyoshi Sekizuka, Makoto Katsumi, Kenichiro Ishikawa, Tatsuhiko Hosaka, Hiroaki Baba, Kengo Oshima, Koichi Tokuda, Masatsugu Hasegawa, Yu Kawazoe, Shigeki Kushimoto, Mitsuo Kaku

**Affiliations:** Tohoku University Graduate School of Medicine, Sendai, Japan (H. Kanamori, T. Aoyagi, M. Katsumi, K. Ishikawa, T. Hosaka, H. Baba, K. Oshima, K. Tokuda, M. Hasegawa, Y. Kawazoe, S. Kushimoto, M. Kaku);; National Institute of Infectious Diseases, Tokyo, Japan (M. Kuroda, T. Sekizuka)

**Keywords:** Chromobacterium haemolyticum, environment, bacteria, pneumonia, respiratory infections, whole-genome sequencing, Japan

## Abstract

We report a severe case of *Chromobacterium haemolyticum* pneumonia associated with near-drowning and detail the investigation of the pathogen and river water. Our genomic and environmental investigation demonstrated that river water in a temperate region can be a source of *C. haemolyticum* causing human infections.

*Chromobacterium* is a genus of gram-negative, facultative anaerobic bacteria; application of 16S rRNA gene sequencing into bacterial taxonomy is expanding its species (*1*–*5*). Most *Chromobacterium* infections in humans have been caused by *C. violaceum* (*6*). Recently, exceptionally rare cases of *C. haemolyticum* infections have been described (*2*,*4*,*7*–*9*), but environmental sources of this pathogen have not been well investigated. We describe a case of *Chromobacterium*-associated pneumonia due to near-drowning and environmental investigation of a river site of the near-drowning. We used whole-genome sequencing (WGS) to identify the *Chromobacterium* species causing pneumonia associated with near-drowning and investigate molecular features, including antimicrobial resistance, virulence, and genetic relatedness of clinical and environmental isolates of *C. haemolyticum*.

## The Study

This study was approved by the institutional review board of Tohoku University Graduate School of Medicine (IRB no. 2018-1-716). In June 2018, a man in his 70s was transported to our emergency center. He had altered consciousness and hypothermia at admission. He had fallen down a bank and into a river in the Tohoku region of Japan while intoxicated from alcohol and was found immersed in the river. He had respiratory failure and required intubation and mechanical ventilation. He had multiple fractures and a cervical cord injury. He had a history of hypertension, diabetes, and benign prostatic hyperplasia but was not immunodeficient. We diagnosed severe aspiration pneumonia and sepsis and treated the patient empirically with intravenous meropenem plus levofloxacin. We detected a nonpigmented, β-hemolytic gram-negative bacillus from both sputum and blood cultures. *C. violaceum* was identified by a matrix-assisted laser desorption/ionization time-of-flight mass spectrometry (VITEK MS; bioMérieux, https://www.biomerieux.com) with a confidence value of 99.9%. We changed the antimicrobial drug regimen to intravenous ceftazidime plus levofloxacin based on antimicrobial susceptibility testing pattern (Appendix 1 Tablef). After 3 weeks of intravenous therapy and critical care, the patient showed clinical improvement and had negative blood and sputum cultures. He was transferred to a community hospital for further rehabilitation and completed an additional 2 months of oral levofloxacin. 

In mid-August, we conducted an environmental investigation of the river water in the area where the patient was found. We collected 500 mL samples of river water, 2 samples at the site where the patient was found and 1 sample 4 km upstream, where he likely fell into the river. We filtered samples through a polyethersulfone filter membrane with a pore size of 0.22 μm. We placed the membrane filters on sheep blood agar plates and incubated for 24 hours at 35°C. We recovered a nonpigmented, β-hemolytic colony similar to clinical isolates from each of the cultures, which we identified as *C. violaceum*. We performed antimicrobial susceptibility testing by using a MicroScan WalkAway 96 plus (Beckman Coulter, https://www.beckmancoulter.com; Appendix 1) and assessed antimicrobial susceptibility patterns of *Chromobacterium* isolates (Appendix 1 Table).

**Figure 1:**
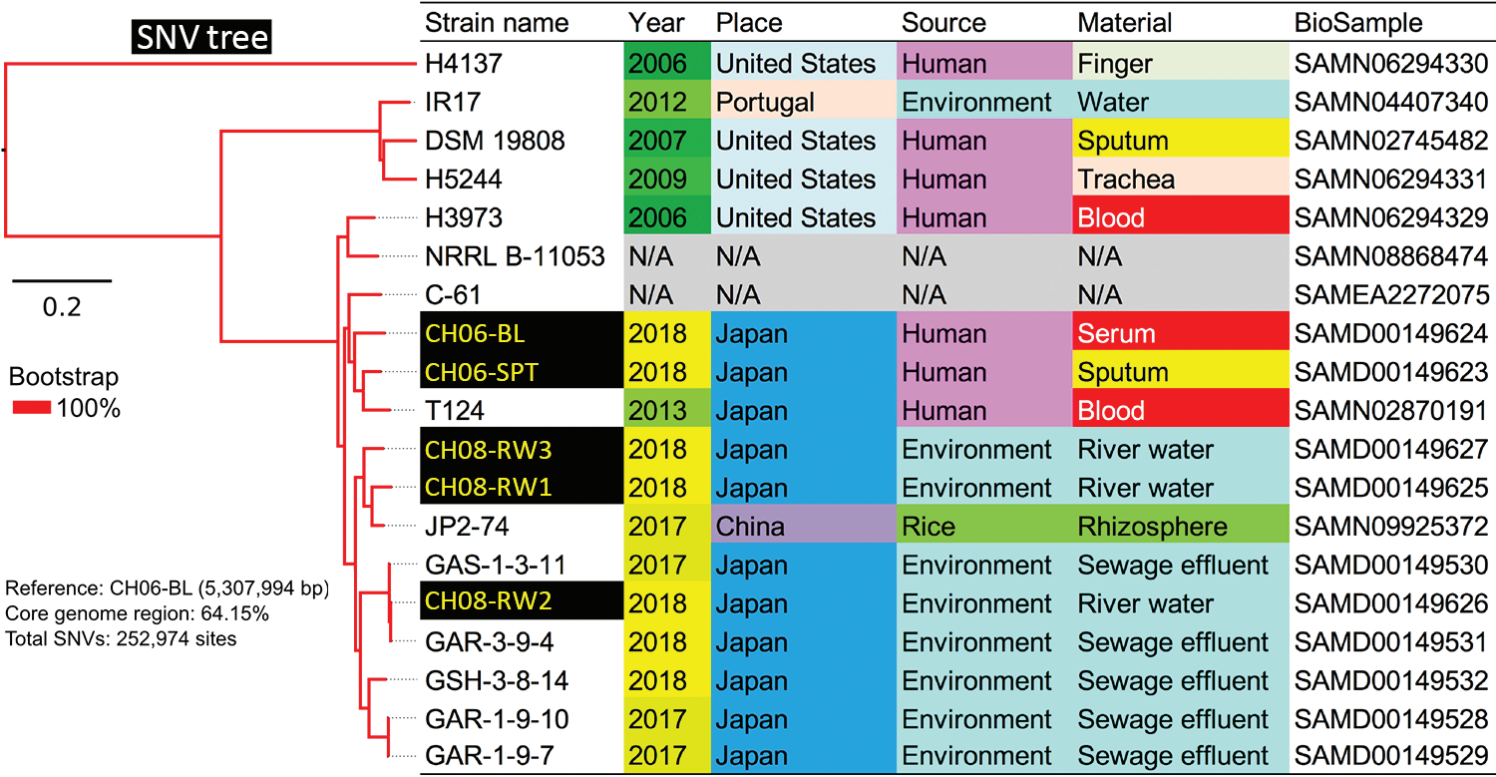
Core genome single-nucleotide variations in a phylogenetic analysis of 19 strains of *Chromobacterium haemolyticum* in a case of pneumonia associated with near-drowning in river water, Japan. In total, 252,974 SNV sites were detected in core genome region among 19 strains. The phylogenetic analysis with SNV data was constructed by maximum likelihood method. Two clinical isolates (CH06-BL and CH06-SPT) and 3 environmental isolates (CH08-RW1, CH08-RW2, and CH08-RW3) of *C. haemolyticum* in this study were discordant (27,867–29,491 SNVs). Scale bar indicates nucleotide substitutions per site. SNV, single nucleotide variant.

**Figure 2:**
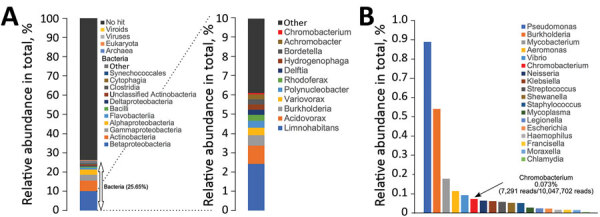
Metagenomic analysis of river water sample collected from the site of near-drowning of a patient with *Chromobacterium haemolyticum* pneumonia, Japan. A) Relative abundance of superkingdom, class of bacteria, and genus of betaproteobacteria in river water sample. The relative abundance of bacteria is 25.65%; the 10 most observed class and genus are summarized in cumulative bar charts. B) Comparison of relative abundance of bacteria causing pneumonia associated with drowning in genus level in the river water sample. The relative abundance of *Chromobacterium*, a Betaproteobacteria, is 0.073%.

We performed WGS on the 3 environmental and 2 clinical isolates (Appendix 1). For comparative genomic analysis, we used additional 16 genome sequences of *Chromobacterium* spp. from wastewater treatment plants in Tokyo and 52 publicly available genome sequences of *Chromobacterium* spp. from the NCBI Assembly database (https://www.ncbi.nlm.nih.gov/assembly) (Figure 1; Appendix 1; Appendix 2 Table 1). We identified 19 strains of *C. haemolyticum* with 252,974 single-nucleotide variants by core-genome phylogenetic analysis (Figure 1; Appendix 2 Table 2). Metagenomic analysis of a river water sample collected from the site of the patient’s near-drowning revealed that the relative abundance of *Chromobacterium* is 0.07% (Figure 2). We deposited the complete genomic sequence of *C. haemolyticum* CH06-BL in GenBank (accession no. AP019312).

## Conclusions

This severe case of drowning-associated pneumonia and bacteremia due to *C. haemolyticum* was successfully treated with appropriate antimicrobial therapy. Previously, 5 clinical cases of *C. haemolyticum* infections had been reported, including sputum colonization, necrotizing fasciitis with bacteremia, proctocolitis, pneumonia, and pediatric bacteremia (*2*,*4*,*7*–*9*). All patients, including the patient we report, survived after antimicrobial treatment. Intravenous antimicrobial therapy, such as meropenem or fluoroquinolone, is recommended for *C. haemolyticum* infections (*7*,*9*). The role of prolonged therapy for *C. haemolyticum* infections remains unclear, but in *C. violaceum* infections, an oral agent such as trimethoprim-sulfamethoxazole, tetracycline, or fluoroquinolone for 2–3 months can be used to prevent relapse (*6*).

As seen in the case we report, identification of *Chromobacterium* species is challenging. *C. violaceum* can produce a violet pigment (violacein) in most strains, and nonpigmented strains rarely have been described (*10*). *C. haemolyticum* does not produce violacein and is characterized by strong hemolytic activity on sheep blood agar (*2*,*4*). Only *C. violaceum* is currently available in the genus *Chromobacterium* on the mass spectrometry database of species identification. Differentiation between *C. haemolyticum* and *C. violaceum* is crucial because *C. haemolyticum* has greater resistance to antimicrobial drugs, such as β-lactams (*2*,*7*). Although *C. aquaticum* is a nonpigmented, β-hemolytic strain phenotypically similar to *C. haemolyticum*, 16S rRNA sequencing might not determine either *C. haemolyticum* or *C. aquaticum* because of artificial separation of both species (*4*). Thus, WGS is a useful tool for accurate identification of *Chromobacterium* species to avoid misidentification of *C. haemolyticum* (*1*–*5*). 

*C. haemolyticum* CH06-BL and other clinical and environmental isolates in this study possessed *bla*_CRH-1_ in the chromosome (Appendix 2 Table 1), but we did not identify mobile elements in the surrounding area. In a previous study, a class A β-lactamase, CRH-1 from *C. haemolyticum* was closely related to *Klebsiella pneumoniae* carbapenemase 2 (*11*). As seen in acquired resistance among other gram-negative bacilli, aquatic environments can be a reservoir (*11*,*12*). 

The etiology of infections caused by *Chromobacterium* has not been fully elucidated. Of note, *Chromobacterium* accounted for only a small portion of the bacteria found in our metagenomics analysis of the river water, but this organism was isolated from the patient and was involved in human infection, despite presence of other potential pathogens in the river, such as *Pseudomonas*, *Aeromonas*, *Legionella*, that can cause pneumonia associated with drowning (Figure 2) (*13*). Our study isolates also had type III secretion system (T3SS) encoded by *Chromobacterium* pathogenicity island 1 and 1a (Cpi-1/-1a) (Appendix 1 Figure 2), which is known as a major virulence factor in *C. violaceum* (*14*). These results highlight the need for further research on antimicrobial resistance and virulence in *Chromobacterium* spp.

*C. violaceum* is widely distributed in natural aquatic environments and can be observed in water and soil sources, especially in tropical and subtropical areas (*6*). *C. haemolyticum* strains with genetic heterogeneity have been detected from lake water in a tropical region (*15*), but the bacterium’s habitat in temperate regions remains unknown. Our comparative genomic analysis revealed that clinical and environmental isolates of *C. haemolyticum* were discordant (27,867–29,491 single-nucleotide variants), although there was no standard definition for its clonality. 

Only 2 reports of cases with *C. haemolyticum* infections in temperate regions of Japan have been published (*7*,*9*). One study reported necrotizing fasciitis associated with exposure to river water after injury. The other described pneumonia caused by accidental aspiration of runoff water after a fall in a ditch and identification of the pathogen in the water and discordant results with clinical isolates by pulsed-field gel electrophoresis. However, detailed environmental investigations of the rivers as a source of the pathogen were not conducted in either article. 

In summary, our genomic and environmental study demonstrates that *C. haemolyticum* in a local river, a natural habitat of this pathogen in Japan, caused this human infection. Clinicians should remain aware that river water in temperate regions can be a source of *C. haemolyticum* infection.

Appendix 1 Additional information and methods used for antimicrobial susceptibility testing and whole-genome sequencing of *Chromobacterium haemolyticum* collected from a patient with pneumonia and river water at the site of near-drowning, Japan.

Appendix 2 *Chromobacterium* spp. used for whole-genome sequencing and comparison in a case of *C. haemolyticum* pneumonia, Japan.
