# Supplementary material for: Chromobacterium haemolyticum Pneumonia Associated with Near-Drowning and River Water, Japan
Source: Emerg Infect Dis. 2020 Sep;26(9):2186–9. doi: 10.3201/eid2609.190670 (PMC7454080; doi:10.3201/eid2609.190670)
Supplement: Appendix 1 — Additional information and methods used for antimicrobial susceptibility testing and whole-genome sequencing of Chromobacterium haemolyticum collected from a patient with pneumonia and river water at the site of near-drowning, Japan. [file 19-0670-Techapp-s1.pdf]

Article DOI: https://doi.org/10.3201/eid2609.190670

Chromobacterium haemolyticum Pneumonia Associated with Near-Drowning and River Water, Japan

Appendix 1

Methods

Antimicrobial Susceptibility Testing

Antimicrobial susceptibility testing of clinical and environmental isolates of *Chromobacterium haemolyticum* was performed by using a MicroScan WalkAway 96 plus (Beckman Coulter, https://www.beckmancoulter.com). Antimicrobial agents tested were as follows: ampicillin/sulbactam, piperacillin, piperacillin/tazobactam, ceftazidime, cefepime, cefozopran, cefoperazone/sulbactam, imipenem, meropenem, amikacin, gentamycin, tobramycin, minocycline, levofloxacin, ciprofloxacin, fosfomycin, aztreonam, chloramphenicol, and trimethoprim-sulfamethoxazole. The breakpoints for each antimicrobial drug were interpreted according to the 2016 Clinical and Laboratory Standards Institute guidelines (CLSI M100-S26; https://www.clsi.org).

Whole-Genome Sequencing

The genomic DNA library of all *Chromobacterium* spp. were constructed by using QIAseq FX DNA Library Kit (Qiagen, https://www.qiagen.com) according to the manufacturer’s instructions, then by paired-end sequencing using an Illumina NextSeq 500 platform with a 300-

cycle NextSeq 500 reagent kit v2 (Illumina, https://www.illumina.com). The metagenomic samples were sequenced by single-end sequencing by using 150-cycle NextSeq 500 Reagent Kit v2 (Illumina). The complete genome sequence of the strain was determined by using a PacBio Sequel (Pacific BioSciences, https://www.pacb.com) sequencer with Sequel SMRT Cell 1M v2 (four/tray) and Sequel sequencing kit v2.1 (Pacific BioSciences) for long-read sequencing (insert size, ≈10 kb). High quality genomic DNA was used to prepare a SMRTbell library by using a SMRTbell template prep kit 2.0 (Pacific Biosciences).

de novo Assembly and Annotation

The draft genome contigs were assembled by using A5-Miseq software version 20140604 with Illumina short reads (*1*). The circular genome sequence was constructed by using Canu version 1.4 (*2*), minimap version 0.2-r124 (*3*), racon version 1.1.0 (*4*), and Circlator version 1.5.3 (*5*) with long read data. Error correction of circular sequence was performed by using Pilon version 1.18 with short reads (*6*). Annotation was performed in DFAST version 1.0.8 (*7*) and NCBI-BLASTP/BLASTX against deposited *Chromobacterium* complete genome sequences.

in silico Genomic and Metagenomic Analysis

For comparative genomic analysis, we downloaded 52 publicly available genome sequences of *Chromobacterium* spp. from NCBI Assembly database (https://www.ncbi.nlm.nih.gov/assembly) (Appendix 1 Table). The species prediction was performed by using average nucleotide identity (ANI) with FastANI program version 1.1 (*8*), *rpoB* phylogenetic analysis with FastTree2 (*9*), and 16S rRNA gene identity search by using BLASTN (*10*) with 16S rRNA reference sequences of 12 *Chromobacterium* strains. The simulated 150 mer paired-end short reads were generated from the available genomic sequences by using SimSeq software (*11*). All short read data was mapped by using bwa-MEM program

(*12*) against the *C. haemolyticum* CH06-BL complete genome sequence (accession no. AP019312) as a reference and single nucleotide variation (SNV) sites were extracted by using VarScan v2.3.4 (*13*). The repeat regions of CH06-BL genomic sequences were predicted by using NUCmer (*14*) and prophage regions were predicted by using PHASTER (*15*; SNVs on these regions were excluded. An SNV phylogenetic tree was constructed by the approximate maximum-likelihood method by using FastTree 2 (*9*), and visualized by using Figtree version 1.4.3 (http://tree.bio.ed.ac.uk/software/figtree).

To characterize the genomic features of *C. haemolyticum* CH06-BL, we performed a BLAST atlas analysis by using GView (*16*) and GView Server (https://server.gview.ca). We confirmed the organism classification of metagenomic sequences by using Centrifuge version 1.0.4 (*17*) with custom database that was built from nt database and RefSeq database of genomic sequences of bacteria, archaea, viruses, and humans.

References

1. Coil D, Jospin G, Darling AE. A5-miseq: an updated pipeline to assemble microbial genomes from Illumina MiSeq data. Bioinformatics. 2015;31:587–9. PubMed https://doi.org/10.1093/bioinformatics/btu661

2. Koren S, Walenz BP, Berlin K, Miller JR, Bergman NH, Phillippy AM. Canu: scalable and accurate long-read assembly via adaptive *k*-mer weighting and repeat separation. Genome Res. 2017;27:722–36. PubMed https://doi.org/10.1101/gr.215087.116

3. Li H. Minimap and miniasm: fast mapping and de novo assembly for noisy long sequences. Bioinformatics. 2016;32:2103–10. PubMed https://doi.org/10.1093/bioinformatics/btw152

4. Vaser R, Sović I, Nagarajan N, Šikić M. Fast and accurate de novo genome assembly from long uncorrected reads. Genome Res. 2017;27:737–46. PubMed https://doi.org/10.1101/gr.214270.116

5. Hunt M, Silva ND, Otto TD, Parkhill J, Keane JA, Harris SR. Circlator: automated circularization of genome assemblies using long sequencing reads. Genome Biol. 2015;16:294. PubMed https://doi.org/10.1186/s13059-015-0849-0

6. Walker BJ, Abeel T, Shea T, Priest M, Abouelliel A, Sakthikumar S, et al. Pilon: an integrated tool for comprehensive microbial variant detection and genome assembly improvement. PLoS One. 2014;9:e112963. PubMed https://doi.org/10.1371/journal.pone.0112963

7. Tanizawa Y, Fujisawa T, Nakamura Y. DFAST: a flexible prokaryotic genome annotation pipeline for faster genome publication. Bioinformatics. 2018;34:1037–9. PubMed https://doi.org/10.1093/bioinformatics/btx713

8. Jain C, Rodriguez-R LM, Phillippy AM, Konstantinidis KT, Aluru S. High throughput ANI analysis of 90K prokaryotic genomes reveals clear species boundaries. Nat Commun. 2018;9:5114. PubMed https://doi.org/10.1038/s41467-018-07641-9

9. Price MN, Dehal PS, Arkin AP. FastTree 2—approximately maximum-likelihood trees for large alignments. PLoS One. 2010;5:e9490. PubMed https://doi.org/10.1371/journal.pone.0009490

10. Altschul SF, Gish W, Miller W, Myers EW, Lipman DJ. Basic local alignment search tool. J Mol Biol. 1990;215:403–10. PubMed https://doi.org/10.1016/S0022-2836(05)80360-2

11. Earl D, Bradnam K, St John J, Darling A, Lin D, Fass J, et al. Assemblathon 1: a competitive assessment of de novo short read assembly methods. Genome Res. 2011;21:2224–41. PubMed https://doi.org/10.1101/gr.126599.111

12. Li H, Durbin R. Fast and accurate long-read alignment with Burrows-Wheeler transform. Bioinformatics. 2010;26:589–95. PubMed https://doi.org/10.1093/bioinformatics/btp698

13. Koboldt DC, Chen K, Wylie T, Larson DE, McLellan MD, Mardis ER, et al. VarScan: variant detection in massively parallel sequencing of individual and pooled samples. Bioinformatics. 2009;25:2283–5. PubMed https://doi.org/10.1093/bioinformatics/btp373

14. Kurtz S, Phillippy A, Delcher AL, Smoot M, Shumway M, Antonescu C, et al. Versatile and open software for comparing large genomes. Genome Biol. 2004;5:R12. PubMed https://doi.org/10.1186/gb-2004-5-2-r12

15. Arndt D, Grant JR, Marcu A, Sajed T, Pon A, Liang Y, et al. PHASTER: a better, faster version of the PHAST phage search tool. Nucleic Acids Res. 2016;44:W16–21. PubMed https://doi.org/10.1093/nar/gkw387

16. Petkau A, Stuart-Edwards M, Stothard P, Van Domselaar G. Interactive microbial genome visualization with GView. Bioinformatics. 2010;26:3125–6. PubMed https://doi.org/10.1093/bioinformatics/btq588

17. Kim D, Song L, Breitwieser FP, Salzberg SL. Centrifuge: rapid and sensitive classification of metagenomic sequences. Genome Res. 2016;26:1721–9. PubMed https://doi.org/10.1101/gr.210641.116

Appendix 1 Table. Antimicrobial susceptibility patterns of clinical and environmental isolates of *Chromobacterium haemolyticum* associated with near-drowning and river water, Japan*

	Patient samples		Environmental samples		
Antimicrobial drug	CH06-SPT	CH06-BL	CH08-RW1	CH08-RW2	CH08-RW3
Ampicillin/sulbactam	>32/16	>32/16	>32/16	>32/16	>32/16
Piperacillin	>64	>64	>64	64	>64
Piperacillin/tazobactam	≤4	8	16	≤4	≤4
Ceftazidime	≤1	≤1	2	≤1	≤1
Cefepime	2	4	8	2	2
Cefozopran	2	4	2	2	2
Cefoperazone/sulbactam	≤8/4	32/16	>32/16	≤8/4	≤8/4
Imipenem	4	>8	>8	4	2
Meropenem	≤0.5	2	4	≤0.5	≤0.5
Amikacin	>32	>32	>32	>32	>32
Gentamycin	>8	>8	>8	8	8
Tobramycin	>8	>8	>8	8	>8
Minocycline	≤1	≤1	4	≤1	4
Levofloxacin	≤0.5	≤0.5	≤0.5	≤0.5	≤0.5
Ciprofloxacin	≤0.25	≤0.25	≤0.25	≤0.25	≤0.25
Fosfomycin	>16	>16	>16	>16	>16
Aztreonam	2	4	2	2	2
Chloramphenicol	≤8	≤8	≤8	≤8	≤8
Trimethoprim-sulfamethoxazole	≤1/19	≤1/19	≤1/19	≤1/19	≤1/19

*Patient samples were collected from sputum and blood; environmental samples were collected from the river at the site of the patient's near-drowning.

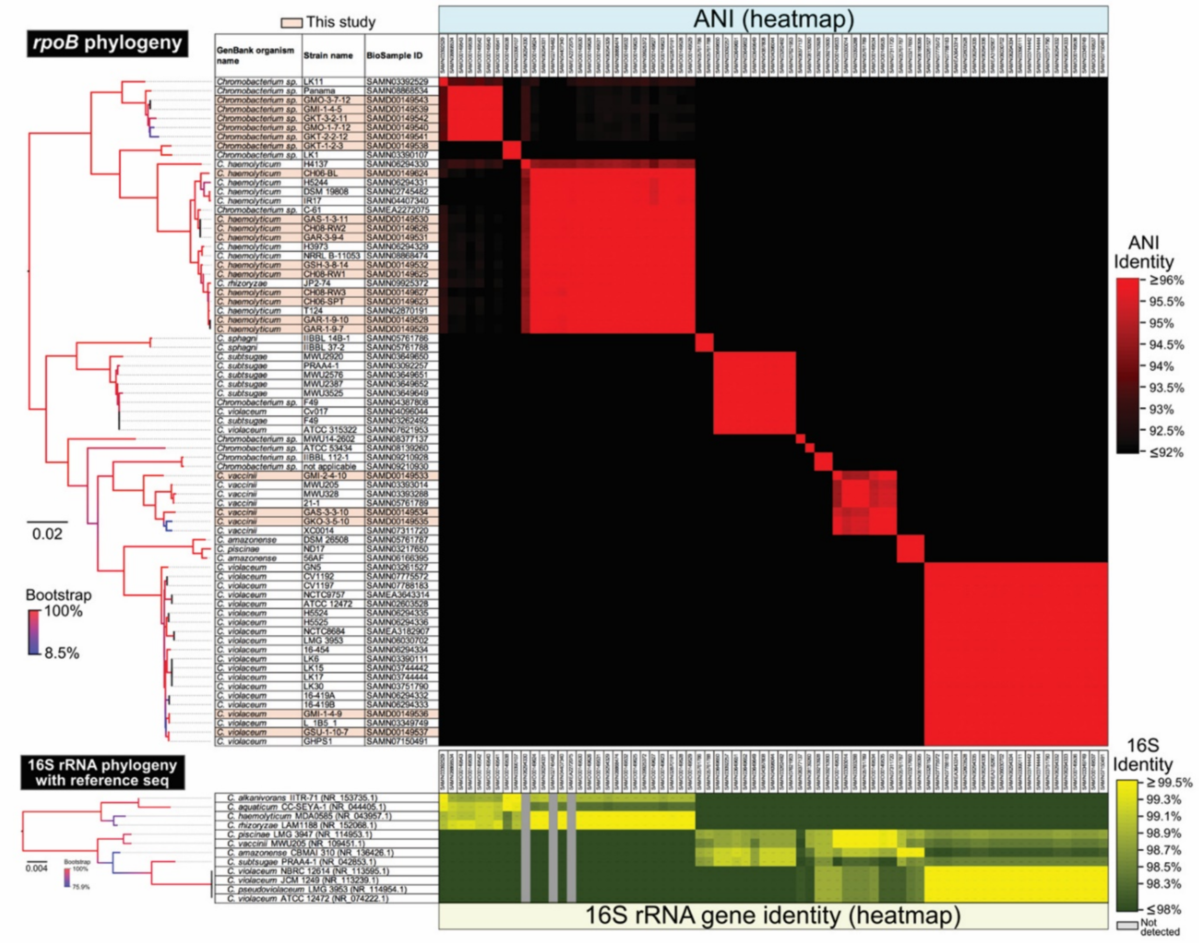

Appendix Figure 1. Heatmap of 16S rRNA of *Chromobacterium haemolyticum* in a case of pneumonia associated with near-drowning in river water, Japan. In total, 252,974 SNV sites were detected in core genome region among 19 strains. The phylogenetic analysis with SNV data was constructed by maximum likelihood method. Two clinical isolates (CH06-BL and CH06-SPT) and 3 environmental isolates (CH08-RW1, CH08-RW2, and CH08-RW3) of *C. haemolyticum* in this study were discordant (27,867–29,491 SNVs). Scale bar indicates nucleotide substitutions per site. SNV, single nucleotide variation.

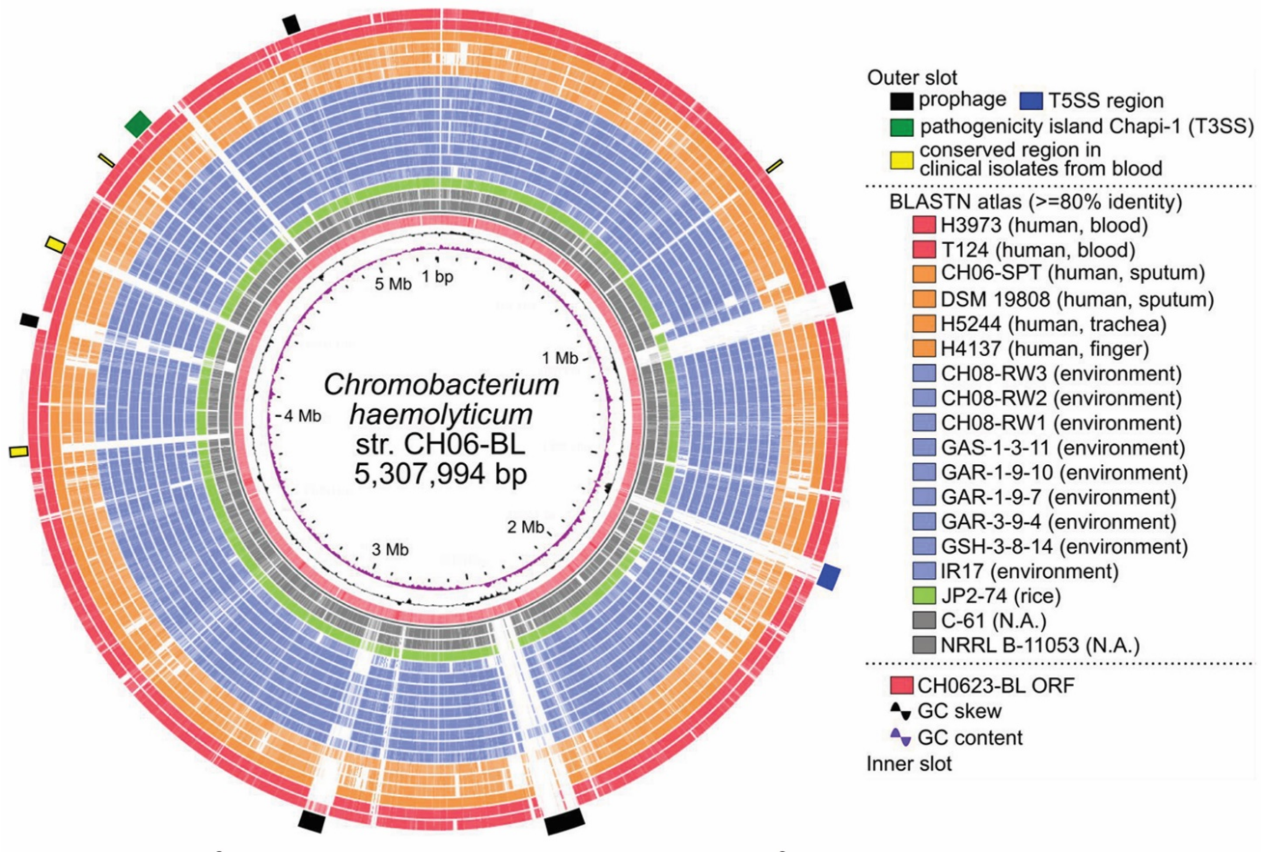

Appendix Figure 2. Comparative genomic analysis among 19 strains of *Chromobacterium haemolyticum* in a case of pneumonia associated with near-drowning in river water, Japan**.** A complete chromosomal sequence of CH06-BL was determined by *de novo* assembly with short- and long-read data, followed by comparison using BLASTatlas analysis between strain CH06-BL and 18 *C. haemolyticum* strains. High homology ($\geq$80% nucleotide identity) regions against CH06-BL chromosome are displayed in each sample slot. Outer slot indicates genomic feature in CH06-BL chromosome; 4 genomic regions are conserved in clinical isolates from blood (yellow labels on outer slot). T3SS, type III secretion system; T5SS, type V secretion system.
